# Supplementary material for: The encephalomyocarditis virus Leader promotes the release of virions inside extracellular vesicles via the induction of secretory autophagy
Source: Nat Commun. 2022 Jun 24;13:3625. doi: 10.1038/s41467-022-31181-y (PMC9232559; doi:10.1038/s41467-022-31181-y)
Supplement: Supplementary file 1 — Supplementary information [file 41467_2022_31181_MOESM1_ESM.pdf]

Supplementary information

The encephalomyocarditis virus Leader promotes the release of virions inside extracellular vesicles via the induction of secretory autophagy

Susanne G. van der Grein[1,4], Kyra A.Y. Defourny[1,4], Huib H. Rabouw[2], Soenita S. Goerdayal[3], Martijn J.C. van Herwijnen[1], Richard W. Wubbolts[1], Maarten Altelaar[3], Frank J.M. van Kuppeveld[2], Esther N.M. Nolte-'t Hoen[1*].

[1]Division of Cell Biology, Metabolism & Cancer, Department of Biomolecular Health Sciences, Faculty of Veterinary Medicine, Utrecht University, Yalelaan 2, 3584 CM Utrecht, The Netherlands.
[2]Virology Section, Division Infectious Diseases & Immunology, Department of Biomolecular Health Sciences, Faculty of Veterinary Medicine, Utrecht University, Yalelaan 1, 3584 CL Utrecht, The Netherlands.
[3]Biomolecular Mass Spectrometry and Proteomics, Bijvoet Center for Biomolecular Research and Utrecht Institute for Pharmaceutical Sciences, Utrecht University, Padualaan 8, 3584 CH Utrecht, The Netherlands.
[4]These authors contributed equally

*Correspondence should be addressed to:

e.n.m.nolte@uu.nl

Supplementary table 1: Proteins identified in EVs by LC-MS/MS

Proteins shared between EMCV-Wt & EMCV-L^{Zn} EVs (n=190)

Accession	Gene	Accession	Gene	Accession	Gene	Accession	Gene
P60709	ACTB	Q02413	DSG1	P32004	L1CAM	P35241	RDX
Q562R1	ACTBL2	P15924	DSP	P11279	LAMP1	P61586	RHOA
P68032	ACTC1	P60981	DSTN	P00338	LDHA	P62913	RPL11
P12814	ACTN1	P68104	EEF1A1	P07195	LDHB	P35268	RPL22
O43707	ACTN4	P13639	EEF2	P49257	LMAN1	P61353	RPL27
P02768	ALB	P06733	ENO1	P02788	LTF	P62888	RPL30
P04075	ALDOA	P15311	EZR	P61626	LYZ	P18077	RPL35A
P09972	ALDOC	P00742	F10	P20645	M6PR	P18124	RPL7
P04083	ANXA1	Q5D862	FLG2	P29966	MARCKS	P62263	RPS14
P50995	ANXA11	Q16658	FSCN1	P49006	MARCKSL1	P60866	RPS20
P07355	ANXA2	P06241	FYN	P43121	MCAM	P23396	RPS3
P08758	ANXA5	P04406	GAPDH	P40925	MDH1	P62070	RRAS2
P02649	APOE	O14556	GAPDHS	P14174	MIF	P31949	S100A11
P02749	APOH	P50395	GDI2	P26038	MSN	P06703	S100A6
P02656	APOC3	P63096	GNAI1	P60660	MYL6	Q8WTV0	SCARB1
P61204	ARF3	P29992	GNA11	Q12965	MYO1E	P01008	SERPINC1
P18085	ARF4	Q14344	GNA13	P15531	NME1	P05546	SERPIND1
P05089	ARG1	P04899	GNAI2	Q9Y639	NPTN	P31947	SFN
P52565	ARHGDIA	P08754	GNAI3	P01111	NRAS	P53985	SLC16A1
P00966	ASS1	P50148	GNAQ	Q9UKS6	PACSIN3	O15427	SLC16A3
P05023	ATP1A1	P63092	GNAS	Q9Y6V0	PCLO	Q15758	SLC1A5
P13637	ATP1A3	P62873	GNB1	O75340	PDCD6	P11166	SLC2A1
P05026	ATP1B1	Q9HAV0	GNB4	Q8WUM4	PDCD6IP	P11169	SLC2A3
P54709	ATP1B3	Q9UBI6	GNG12	P30101	PDIA3	P08195	SLC3A2
P20020	ATP2B1	P06744	GPI	P07737	PFN1	Q8NBI5	SLC43A3
Q01814	ATP2B2	Q8NFJ5	GPRC5A	P18669	PGAM1	Q01650	SLC7A5
P25311	AZGP1	P69905	HBA1	P00558	PGK1	P27105	STOM
P80723	BASP1	P68871	HBB	O43175	PHGDH	Q16563	SYPL1
P35613	BSG	P01891	HLA-A	P14618	PKM	P02786	TFRC
Q10589	BST2	P30493	HLA-B	Q06830	PRDX1	P60174	TPI1
P62158	CALM1	P30508	HLA-C	P32119	PRDX2	Q12931	TRAP1
P31944	CASP14	P07910	HNRNPC	Q13162	PRDX4	P02766	TTR
Q5ZPR3	CD276	P32754	HPD	Q9P2B2	PTGFRN	Q9BQE3	TUBA1C
P16070	CD44	Q86YZ3	HRNR	P61026	RAB10	P68366	TUBA4A
P60033	CD81	P07900	HSP90AA1	P62491	RAB11A	P07437	TUBB
P23528	CFL1	P08238	HSP90AB1	P51153	RAB13	P68371	TUBB4B
Q9Y281	CFL2	P14625	HSP90B1	P59190	RAB15	Q9BUF5	TUBB6
P06732	CKM	P0DMV8	HSPA1A	Q15286	RAB35	P0CG48	UBC
O00299	CLIC1	P11021	HSPA5	P20339	RAB5A	Q5T750	XP32
P09543	CNP	P11142	HSPA8	P51148	RAB5C	Q9NQH7	XPNPEP3
P02452	COL1A1	P04792	HSPB1	P51149	RAB7A	P07947	YES1
P00450	CP	P05362	ICAM1	P63000	RAC1	P31946	YWHAB
O75131	CPNE3	P17936	IGFBP3	P11233	RALA	P62258	YWHAE
P01040	CSTA	P06756	ITGAV	P11234	RALB	P61981	YWHAG
P07339	CTSD	P05556	ITGB1	P62826	RAN	Q04917	YWHAH
Q14118	DAG1	P05106	ITGB3	P62834	RAP1A	P27348	YWHAQ
P81605	DCD	P19823	ITIH2	P61224	RAP1B	P63104	YWHAZ
Q08554	DSC1	P14923	JUP				

Unique EMCV-Wt EVs (n=44) **Unique EMCV-L^{Zn} EVs (n=85)**

Accession	Gene	Accession	Gene	Accession	Gene
P09525	ANXA4	P01023	A2M	O00159	MYO1C
P08133	ANXA6	Q9UNQ0	ABCG2	Q92542	NCSTN
P20073	ANXA7	Q7Z5M8	ABHD12B	P06748	NPM1
P15291	B4GALT1	O60488	ACSL4	P09874	PARP1
Q5T0U0	CCDC122	P02647	APOA1	P30086	PEBP1
P08174	CD55	P02652	APOA2	P52209	PGD
P19256	CD58	P23634	ATP2B4	O15031	PLXNB2
P21926	CD9	P06576	ATP5F1B	P62937	PPIA
O43633	CHMP2A	P61769	B2M	Q15365	PCBP1
Q9H444	CHMP4B	P01024	C3	Q9H0U4	RAB1B
Q86YQ8	CPNE8	P27824	CANX	Q86SE5	RALYL
Q9UBR2	CTSZ	P40121	CAPG	P27635	RPL10
P78310	CXADR	P52907	CAPZA1	P50914	RPL14
Q96JB1	DNAH8	P47756	CAPZB	P61313	RPL15
Q969X5	ERGIC1	P12277	CKB	P18621	RPL17
Q7Z5G4	GOLGA7	Q00610	CLTC	Q07020	RPL18
P31025	LCN1	P68400	CSNK2A1	P83731	RPL24
P47929	LGALS7	P00533	EGFR	P46776	RPL27A
Q99732	LITAF	P60842	EIF4A1	P49207	RPL34
Q12907	LMAN2	P13929	ENO3	P36578	RPL4
P07948	LYN	P00734	F2	P62917	RPL8
P20585	MSH3	P02774	GC	P62280	RPS11
Q15181	PPA1	P06396	GSN	P62249	RPS16
P23284	PPIB	P09211	GSTP1	P62851	RPS25
P15151	PVR	P04908	HIST1H2AB	P62854	RPS26
P61106	RAB14	Q96KK5	HIST1H2AH	P61247	RPS3A
P10114	RAP2A	P06899	HIST1H2BJ	P62753	RPS6
Q15293	RCN1	O60814	HIST1H2BK	P62241	RPS8
O75695	RP2	P68431	HIST1H3A	P05109	S100A8
P05388	RPLP0	P62805	HIST1H4A	Q8NC51	SERBP1
P10301	RRAS	P09651	HNRNPA1	P01009	SERPINA1
O14828	SCAMP3	Q00839	HNRNPU	P29508	SERPINB3
O00560	SDCBP	P00738	HP	Q9H2H9	SLC38A1
O75396	SEC22B	P01112	HRAS	O14745	SLC9A3R1
Q96P63	SERPINB12	P34932	HSPA4	Q8IZP2	ST13P4
P50454	SERPINH1	P10809	HSPD1	P61956	SUMO2
O00161	SNAP23	P46940	IQGAP1	P37802	TAGLN2
P30626	SRI	Q06033	ITIH3	P02787	TF
Q12846	STX4	P01591	JCHAIN	Q86YD3	TMEM25
Q92734	TFG	P17931	LGALS3	P06753	TPM3
P22735	TGM1	Q9C099	LRRCC1	P18206	VCL
P49755	TMED10	P40926	MDH2	P04004	VTN
Q9BVK6	TMED9	P35579	MYH9		
P10599	TXN				

Proteins detected in density gradient purified 100K EV preparations from EMCV-Wt and EMCV-L^{Zn} infected cells are listed based on their exclusive or shared identification among the different sample conditions. For protein identification, the MS-generated data was searched against the UniProtKB/Swiss-Prot database (all entries). Proteins listed were identified in at least 2/3 independent replicates.

Supplementary figure 1

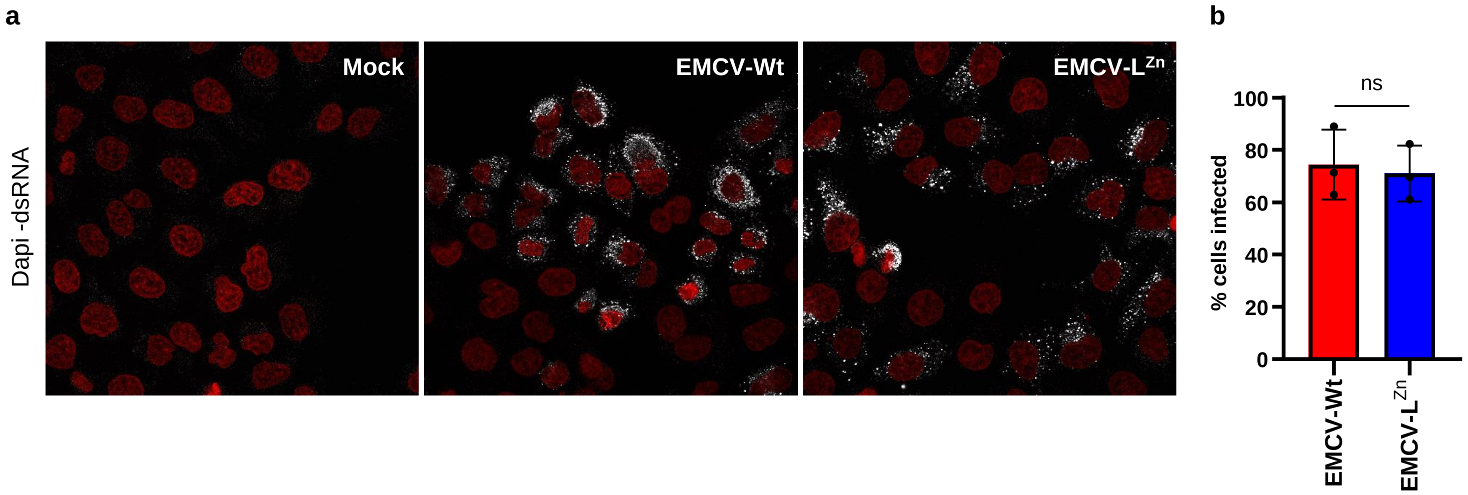

Supplementary figure 1. Infection efficiency of EMCV-Wt *vs.* EMCV-L^{Zn}. Cells were mock infected or infected with EMCV-Wt or EMCV-L^{Zn} at MOI 10. dsRNA staining was performed to identify infected cells 6-8 h p.i. (a) Depicted are representative confocal images, showing DAPI nuclear staining (red) and dsRNA staining (grey). (b) Quantification of the percentage of infected cells in n=3 independent experiments. For each experiment 2-3 images containing 11-42 cells per image were scored and the average infection efficiencies were calculated ± SD. ns, p=0.7497 as determined by a two-tailed t-test. Source data are provided as a Source Data file.

Supplementary figure 2

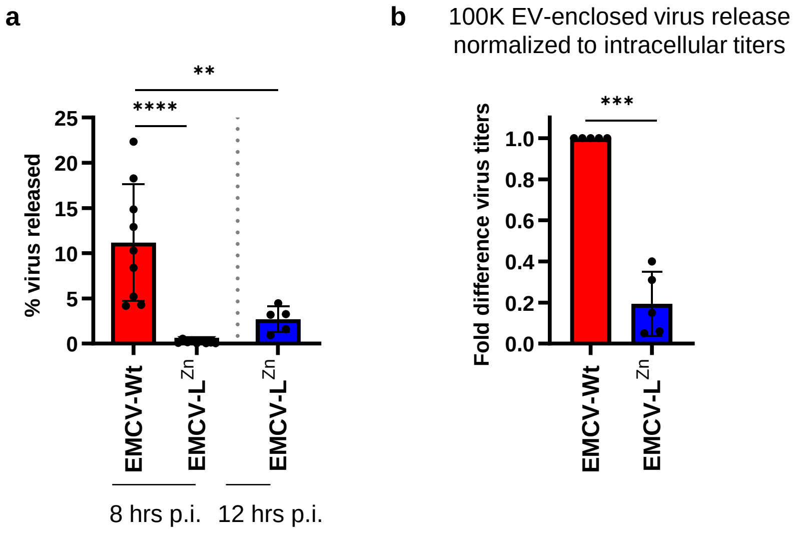

Supplementary figure 2. Non-lytic virus release by EMCV-Wt *vs*. EMCV-L^{Zn} infected cells. (a) Extracellular virus release by EMCV-Wt and EMCV-L^{Zn} infected cells depicted as percentage of the total virus (combined intracellular and extracellular titers) produced at the indicated timepoints. The mean percentages ± SD of n=9 experiments for EMCV-Wt and EMCV-L^{Zn} at 8 hrs p.i. and n=5 experiments for EMCV-L^{Zn} at 12 hrs p.i. are shown. ** p=0.0028, **** p<0.0001, as assessed by one-way ANOVA with Dunnett's multiple comparisons test. (b) EV-enclosed virus release was determined 8 hours p.i. by end-point dilution of density gradient purified EV isolated at 100,000x*g*. Depicted is the mean fold difference in EV-enclosed virus release induced by EMCV-L^{Zn} relative to EMCV-Wt after normalization for intracellular virus titers ± SD (n=5). *** p=0.0003, as assessed by a two-tailed one-sample t-test. Source data are provided as a Source Data file.

Supplementary figure 3

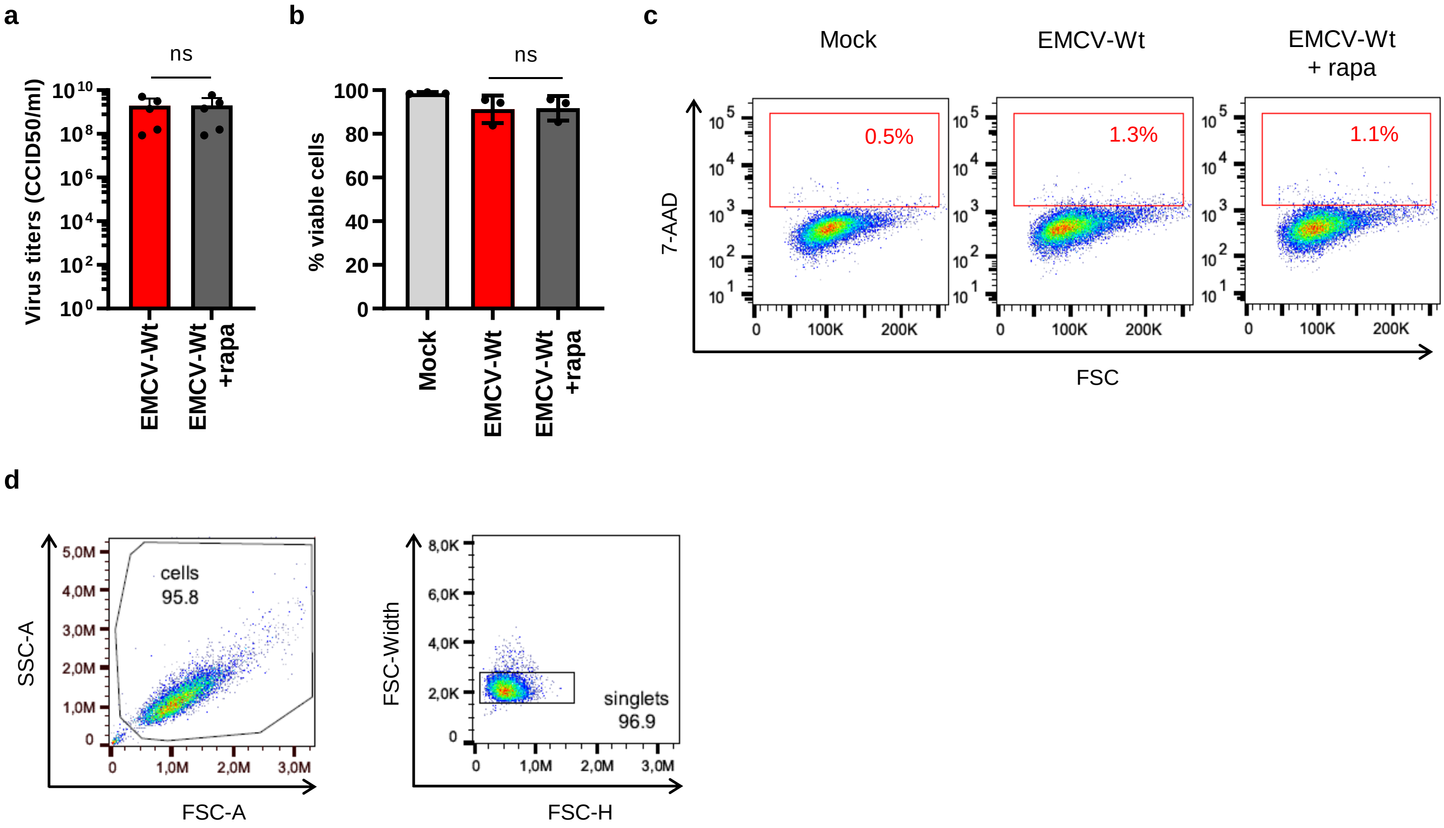

Supplementary figure 3. The impact of rapamycin treatment on virus production and viability of EMCV-Wt infected cells. (a) Cells were infected with EMCV-Wt for 8 hrs and treated or not with rapamycin from 1 hr p.i. onwards, after which intracellular virus production was assessed by end-point dilution. Indicated are mean values ± SD of n=5 individual experiments. ns, p=0.9577 as determined by a two-tailed t-test. (b) Viability of cells treated as in (a) was determined by flow cytometric analysis of fluorescently stained dead cells. The percentage of viable cells is depicted for n=3 independent experiments. ns, p=0.9132 as determined by a two-tailed t-test. (c) Dot plots illustrating the gating strategy to discriminate between live/dead cells. (d) Dots plots illustrating the gating strategy for the selection of single cells prior to viability analysis. Source data are provided as a Source Data file.

Supplementary figure 4

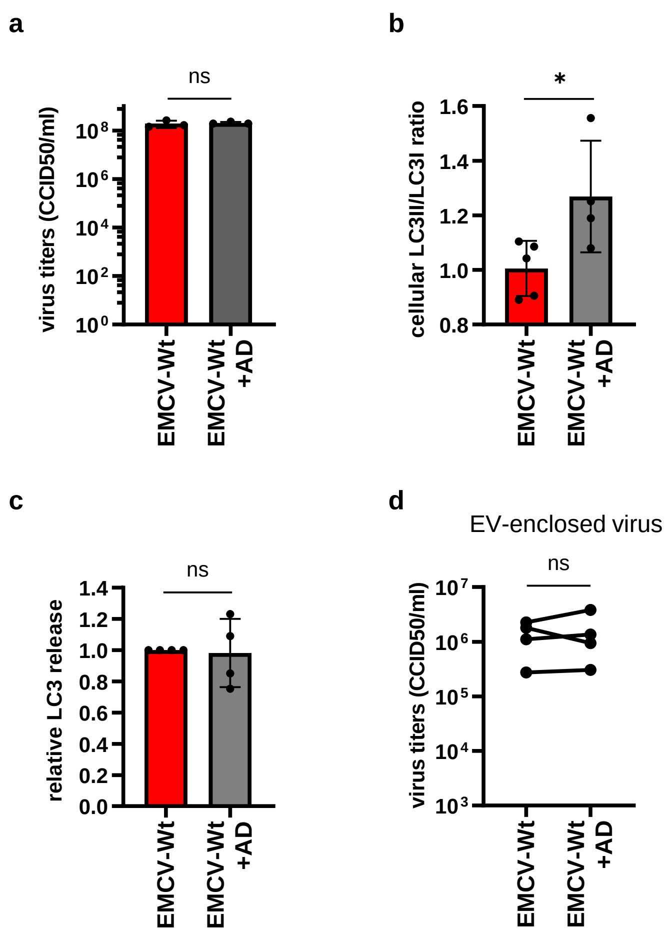

Supplementary figure 4. Treatment of EMCV-Wt infected cells with amiodarone, a known enhancer of autophagic flux. (a) Cells were infected with EMCV-Wt for 8 hrs and treated or not with amiodarone from 1 hr p.i. onwards, after which intracellular virus production was assessed by end-point dilution in n=3 independent samples. ns, p=0.7749 as determined by a two-tailed t-test. (b) LC3-II:LC3-I ratios as detected by western blot were calculated in whole cell lysates from samples treated as in (a) to confirm stimulation of autophagy by amiodarone. * p=0.0380 using a two-tailed t-test for n=4 (EMCV-Wt + AD) or n=5 (EMCV-Wt) independent experiments. (c) LC3 release by EMCV-Wt infected cells in the presence of amiodarone was quantified relative to the untreated control as detected by western blotting in ultracentrifugation pellets prepared from supernatants of equal numbers of cells. ns, p=0.8785 using a two-tailed one-sample t-test for n=4 independent experiments. (d) EV-enclosed virus titers were determined by end-point dilution assay after density gradient-based purification of EVs. ns, p=0.7900 using a two-tailed t-test for n=4 independent experiments. Indicated are mean values ± SD. Source data are provided as a Source Data file.

Supplementary figure 5

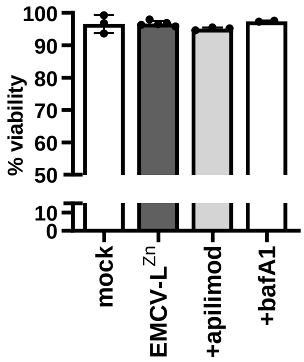

Supplementary figure 5. The impact of apilimod and bafilomycin treatment on the viability of EMCV-L^{Zn} infected cells. EMCV-L^{Zn} infected cells were treated with 500 nM apilimod for 16 hrs prior to infection or 200 nM bafA1 1 hr p.i. onwards. Cell viability, as assessed by live-dead staining and flow cytometric analysis, was compared to untreated EMCV-L^{Zn} infected cells or uninfected controls. Indicated are the mean percentage of viable cells ± SD of n=2 (+bafA1), n=3 (+apilimod, mock), or n=5 (EMCV-L^{Zn}) individual experiments. Gating strategy was identical to that illustrated in Supplementary figure 3c,d. Source data are provided as a Source Data file.

Supplementary figure 6

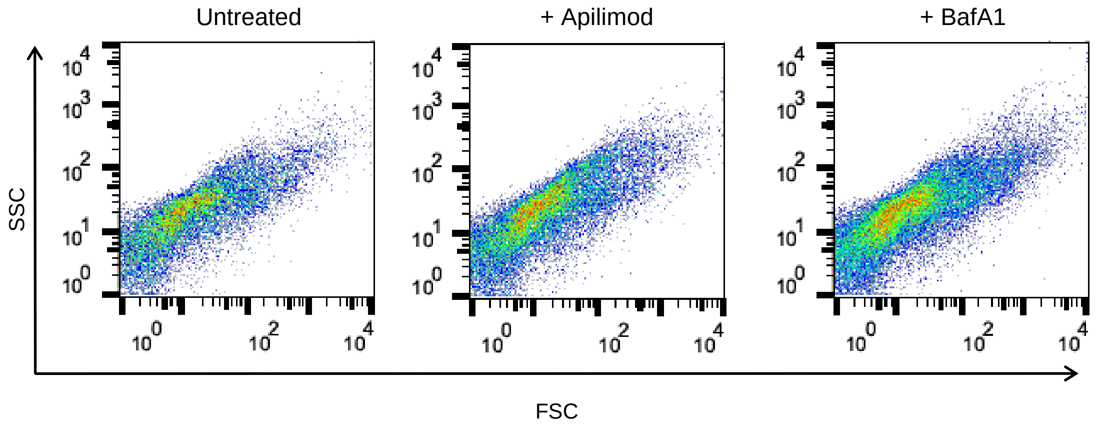

Supplementary figure 6. The light scattering properties of EVs released by EMCV-L^{Zn} infected cells upon treatment with apilimod or bafilomycin. Depicted are representative dot plots of EVs within the 1.08 g/ml density fractions of the samples corresponding to Figure 6b.
