# Supplementary material for: The encephalomyocarditis virus Leader promotes the release of virions inside extracellular vesicles via the induction of secretory autophagy
Source: Nat Commun. 2022 Jun 24;13:3625. doi: 10.1038/s41467-022-31181-y (PMC9232559; doi:10.1038/s41467-022-31181-y)
Supplement: Supplementary file 3 — Reporting Summary [file 41467_2022_31181_MOESM3_ESM.pdf]

nature portfolio

Corresponding author(s): Esther N.M. Nolte-'t Hoen

Last updated by author(s): May 17, 2022

Reporting Summary

Nature Portfolio wishes to improve the reproducibility of the work that we publish. This form provides structure for consistency and transparency in reporting. For further information on Nature Portfolio policies, see our Editorial Policies and the Editorial Policy Checklist.

Statistics

For all statistical analyses, confirm that the following items are present in the figure legend, table legend, main text, or Methods section.

n/a	Confirmed	
☐	☒	The exact sample size (*n*) for each experimental group/condition, given as a discrete number and unit of measurement
☐	☒	A statement on whether measurements were taken from distinct samples or whether the same sample was measured repeatedly
☐	☒	The statistical test(s) used AND whether they are one- or two-sided<br>*Only common tests should be described solely by name; describe more complex techniques in the Methods section.*
☒	☐	A description of all covariates tested
☐	☒	A description of any assumptions or corrections, such as tests of normality and adjustment for multiple comparisons
☐	☒	A full description of the statistical parameters including central tendency (e.g. means) or other basic estimates (e.g. regression coefficient) AND variation (e.g. standard deviation) or associated estimates of uncertainty (e.g. confidence intervals)
☐	☒	For null hypothesis testing, the test statistic (e.g. *F*, *t*, *r*) with confidence intervals, effect sizes, degrees of freedom and *P* value noted<br>*Give P values as exact values whenever suitable.*
☒	☐	For Bayesian analysis, information on the choice of priors and Markov chain Monte Carlo settings
☒	☐	For hierarchical and complex designs, identification of the appropriate level for tests and full reporting of outcomes
☒	☐	Estimates of effect sizes (e.g. Cohen's *d*, Pearson's *r*), indicating how they were calculated

Our web collection on statistics for biologists contains articles on many of the points above.

Software and code

Policy information about availability of computer code

Data collection	Western blot images were collected using Image Lab software (Bio-Rad) (version 5.1). MS raw data were processed with Proteome Discoverer (version 1.4.1.14, Thermo Fisher). Peptide identification was performed using Mascot 2.3 (Matrix Science, UK). Peak lists were generated from the raw data files using Proteome Discoverer version 1.4.1.14 (Thermo Scientific, Bremen) and searched against the UniProtKB/Swiss-Prot database (uniprot.org, consulted in July 2018) (all entries for identification of EMCV proteins, Homo sapiens for identification of host proteins), supplemented with frequently observed contaminants. Flow cytometry data were acquired using FACS Diva software (version 6.1.3).
Data analysis	Protein-protein interactions were analyzed using the STRING database version 11.0 (https://string-db.org/). Functional enrichment analysis was performed using PANTHER (via http://geneontology.org). Graphs were generated and statistically analyzed using Graphpad Prism 8 (Graphpad Software, CA). Flow cytometry data analysis was performed using FlowJo v10.07 (FlowJo 491 LLC, Ashland, OR) or FCS expression v3 (De Novo software, Los Angeles, CA). Confocal images were analyzed using NIS elements 5.1 general analysis module (Nikon 475 Microsystems), or ImageJ software (1.52i).

For manuscripts utilizing custom algorithms or software that are central to the research but not yet described in published literature, software must be made available to editors and reviewers. We strongly encourage code deposition in a community repository (e.g. GitHub). See the Nature Portfolio guidelines for submitting code & software for further information.

Data

Policy information about availability of data

All manuscripts must include a data availability statement. This statement should provide the following information, where applicable:

- Accession codes, unique identifiers, or web links for publicly available datasets
- A description of any restrictions on data availability
- For clinical datasets or third party data, please ensure that the statement adheres to our policy

Source data are provided with this paper. The MS raw data generated in this study have been deposited to the ProteomeXchange Consortium via the PRIDE partner repository (http://www.ebi.ac.uk/pride, under accession code PXD033906). We have submitted all relevant data of our experiments to the EV-TRACK knowledgebase (EV-TRACK ID: EV220089, https://evtrack.org/, under the accession code 'VanderGrein').

Field-specific reporting

Please select the one below that is the best fit for your research. If you are not sure, read the appropriate sections before making your selection.

☒ Life sciences ☐ Behavioural & social sciences ☐ Ecological, evolutionary & environmental sciences

For a reference copy of the document with all sections, see nature.com/documents/nr-reporting-summary-flat.pdf

Life sciences study design

All studies must disclose on these points even when the disclosure is negative.

Sample size	Statistical methods were not used to predetermine sample size. For all samples on which statistical analysis was performed at least n=3 independent experiments were performed to be able to estimate sample variance. In case large sample variance was observed, an additional n=4 replicate was performed to ensure proper interpretation of the results. In case additional experiments were performed for other reasons (for example when additional treatment conditions were assessed in a separate experiment) all data points were included for analysis to ensure data transparancy and reproducibility.
Data exclusions	Data sets involving rapamycin treatment in which due to culture conditions basal autophagy levels in the cells was already increased to the point that no further induction of autophagy could be observed in response to rapamycin (rapamycin-unresponsive) were excluded from analysis.
Replication	All datapoints were replicated in at least two independent experiments, and in the case of statistical analysis at least n=3 independent experiments to ensure reproducibility. For virus infections, in addition at least two independent virus batches were used.
Randomization	No randomization was applied, given the small sample sets and lack of datasets that could be subject to non-objective interpretation. For Supplementary Figure 1, a second independent researcher was asked to verify the scoring to determine that also this dataset was not subjected to any observer bias.
Blinding	No blinding could be performed, as knowledge about the treatment conditions was required for the researchers to be able to perform the experiments and insure proper sample handling.

Reporting for specific materials, systems and methods

We require information from authors about some types of materials, experimental systems and methods used in many studies. Here, indicate whether each material, system or method listed is relevant to your study. If you are not sure if a list item applies to your research, read the appropriate section before selecting a response.

Materials & experimental systems

n/a	Involved in the study	
☐	☒	Antibodies
☐	☒	Eukaryotic cell lines
☒	☐	Palaeontology and archaeology
☒	☐	Animals and other organisms
☒	☐	Human research participants
☒	☐	Clinical data
☒	☐	Dual use research of concern

Methods

n/a	Involved in the study	
☒	☐	ChIP-seq
☐	☒	Flow cytometry
☒	☐	MRI-based neuroimaging

Antibodies

Antibodies used	mouse-α-CD63 (1:1000, clone TS63; catalogue #ab59479, Abcam, Cambridge, UK), mouse-α-CD9 (1:2000, clone HI9a; Biolegend, San

Antibodies used	Diego, CA), mouse-α-Flotillin-1 (1:1000, clone 18/Flotillin-1; catalogue #610820, BD Biosciences), mouse-α-LC3 (1:500, clone 5F10; catalogue #0231-100/LC3-5F10; 1:50, clone 2G6; catalogue #0260-100/LC3-2G6, Nanotools, Teningen, Germany), rabbit-α-LC3 (1:1000, polyclonal; catalogue #PM036, MBL international, Woburn, MA), mouse-α-β actin (1:30,000, clone AC-15, catalogue #A5441, Sigma-Aldrich), mouse-α-EMCV 3D polymerase (1:100, clone 3B7, catalogue #sc-65633, Santa Cruz Biotechnology, Dallas, TX), peroxidase-AffiniPure (HRP-conjugated) Goat-α-mouse IgG + IgM (H+L) (1:10,000, polyclonal; catalogue #115-035-044, Jackson ImmunoResearch Labaratories, lot #116723), goat-α-rabbit Immunoglobulins/HRP (1:2500, polyclonal; catalogue #P0448, DAKO, Denmark, lot #00077731), mouse-α-dsRNA (1:200-1:1000, J2; catalogue #10010500, English Scientific & Consulting, Hungary), donkey-α-mouse Alexa-488 or 647 (1:200, catalogue #A21202 or A31571, Invitrogen, UK).
Validation	Commercial antibodies were validated by the suppliers, we refer to the information on the supplier's websites.<br>- anti-CD63: https://www.abcam.com/cd63-antibody-ts63-bsa-and-azide-free-ab59479.html<br>- anti-CD9: https://www.biolegend.com/en-gb/products/purified-anti-human-cd9-antibody-2211?pdf=true&displayInline=true&leftRightMargin=15&topBottomMargin=15&filename=Purified%20anti-human%20CD9%20Antibody.pdf<br>- anti-Flotillin: https://www.bdbiosciences.com/content/bdb/paths/generate-tds-document.nl.610820.pdf<br>- anti-LC3: https://www.mblintl.com/products/wp-content/uploads/sites/2/2021/02/PM036-v12.pdf; http://www.nanotools.de/datasheets/DS-0231-100-LC3-5F10-08-311013F.pdf; http://www.nanotools.de/datasheets/DS-0260-100-LC3-2G6-09-311013F.pdf<br>- anti-actin: https://www.sigmaaldrich.com/deepweb/assets/sigmaaldrich/product/documents/296/386/a5441dat.pdf<br>- anti-EMCV 3D: https://datasheets.scbt.com/sc-65633.pdf<br>-anti-dsRNA: https://www.nordicmubio.com/products/mouse-anti-double-stranded-rna-j2/10010500<br><br>- goat-anti-mouse IgG + IgM -HRP: https://www.jacksonimmuno.com/catalog/products/115-035-003<br>- goat-anti-rabbit Ig - HRP: https://www.agilent.com/store/productDetail.jsp?catalogId=P044801-2&catId=SubCat3ECS_244796<br>- donkey-anti-mouse Alexa 488: https://www.thermofisher.com/order/genome-database/dataSheetPdf?producttype=antibody&productsubtype=antibody_secondary&productId=A-21202&version=214<br>- donkey-anti-mouse Alexa 647: https://www.thermofisher.com/order/genome-database/dataSheetPdf?producttype=antibody&productsubtype=antibody_secondary&productId=A-31571&version=214<br><br>Additional validation by researchers:<br>- For validation of LC3 antibodies, commercially available cell lysates enriched for LC3I or LC3II (Nanotools, catalogue #1041/PC3/LC3I and #1042/PC3/LC3II) were analyzed. Banding pattern was consistent with that previously reported for the different modification forms of LC3 (Mizushima N., Yoshimori T. How to interpret LC3 immunoblotting. Autophagy 3, 542-5, (2007)).<br>- For the anti-EMCV 3D and the anti-dsRNA antibody uninfected cells were taken along in the same experiment.

Eukaryotic cell lines

Policy information about cell lines

Cell line source(s)	Human cervical carcinoma cells (HeLa R19) were a kind gift from Dr G. Belov (University of Maryland, 408 USA). These cells are not commercially available. Baby hamster kidney cells (BHK21, ATTC CCL-10) were obtained from the American Type 409 Culture Collection (Rockville, MD)
Authentication	None of the cell lines were authenticated
Mycoplasma contamination	All cell lines used were tested negative for mycoplasma contamination
Commonly misidentified lines<br>(See ICLAC register)	no commonly misidentified cell lines were used

Flow Cytometry

Plots

Confirm that:

☒ The axis labels state the marker and fluorochrome used (e.g. CD4-FITC).

☒ The axis scales are clearly visible. Include numbers along axes only for bottom left plot of group (a 'group' is an analysis of identical markers).

☒ All plots are contour plots with outliers or pseudocolor plots.

☒ A numerical value for number of cells or percentage (with statistics) is provided.

Methodology

Sample preparation	Cell viability was assessed using 7-AAD viability staining (eBioscience, San Diego, CA) or Fixable Viability Dye eFluor™ 506 (eBioscience, San Diego, CA) according to manufacturer's protocols. In short, after infection adherent HeLa-R19 cells were harvested by trypsinization and pooled with any detached cells recovered from the supernatant following centrifugation at 200xg for 10 min. For 7-AAD staining cells were washed with phosphate buffered saline (PBS), and stained with 5 µL dye per 1x10^6 cells for 5 min at RT. For the Fixable Viability Dye eFluor™ 506 labelling, cells were washed twice in ice cold PBS followed by staining on ice for 30 min using a 1:1000 working dilution. Unbound dye was removed by washing with PBS and the cells were fixed in 1% paraformaldehyde (PFA). For the detection of extracellular vesicles (EVs), EVs were isolated from cell culture supernatants containing 10% EV-depleted FCS (pelleted for >16 hrs at 100,000xg and passaged through a 0.22 micrometer filter). To this end, samples were cleared of cells and debris by centrifugation 1-2x at 200xg for 10 min, 2x at 500xg for 10 min, and 1x 10,000xg for 30 min. EV were pelleted for 65 min at 100,000xg, resuspended in 0.1-0.2% exofree

BSA/PBS and next labeled with 30 µM CFSE (Invitrogen, Carlsbad, CA) in 50 µl PBS for 1 hr at RT. Unbound CFSE was separated from EVs by density gradient centrifugation. EVs in gradient fractions were fixed with 2% paraformaldehyde for 30 min and diluted in PBS.

Instrument	FACS-based viability assays were performed on a CytoFLEX LX (Beckman Coulter) for cells labeled with fixable Viability Dye eFluor™ 506 or a BD FACS Canto II (BD Biosciences, San Jose, CA) with BD FACS Diva software for cells labeled with 7-AAD viability staining. High resolution flow cytometric analysis of EVs was done using a BD Influx flow cytometer with optimized configuration, as described previously (Nolte-'t Hoen et al. Nanomedicine 2012, Van der Vlist et al. Nature protocols 2012). In short, thresholding was applied on fluorescence generated by CFSE-labeled EVs passing the 488 nm laser. Fluorescent 100 nm and 200 nm polystyrene beads (FluoSpheres, Invitrogen) were used to calibrate the fluorescence, and forward (FSC) and side (SSC) scattered light settings. Samples were measured at low pressure (sheath fluid: 5 PSI, sample: 4.2 PSI) using a 140 micrometer nozzle with event rates below 10,000 per second.
Software	Data was gathered using the software accompanying the indicated machines. For data analysis, FlowJo v10.07 software (FlowJo, Ashland, OR, USA) or FCS expression v3 (De Novo software, Los Angeles, CA) was used.
Cell population abundance	10,000 events were recorded for FACS-based viability analysis, ensuring a minimum of 6000 single cells for quantification. For high resolution flow cytometric analysis of EVs, samples were measured in a fixed time window (30s) to allow direct comparison of EV concentrations in parallel samples. A PBS control was taken along to ensure that sufficient EVs could be detected above background.
Gating strategy	For cell viability analysis, cells were gated based on SSC-A vs. FSC-A plots. Single cells were subsequently selected using a FSC-H vs. FSC-width gate. Viable cells were finally gated based on FSC-A vs. AAD-7 Viability dye/Fixable viability dye eFluor 506 plots. To determine the boundaries between living/dead cells and the success of labelling, a mix of heat shocked cells (3 min 42 degrees, followed by 1 min on ice) and untreated cells was taken along as positive control. In addition, unstained cells were measured as negative control. EVs measured by high resolution flow cytometry were divided in FSC-high or FSC-low EV subsets based on SSC-A vs FSC-A plots, using pseudocolored dot plots to determine the boundaries between the two respective populations for each respective experiment.

☒ Tick this box to confirm that a figure exemplifying the gating strategy is provided in the Supplementary Information.
